# *Enterococcus faecium*: from microbiological insights to practical recommendations for infection control and diagnostics

**DOI:** 10.1186/s13756-020-00770-1

**Published:** 2020-08-10

**Authors:** Xuewei Zhou, Rob J. L. Willems, Alexander W. Friedrich, John W. A. Rossen, Erik Bathoorn

**Affiliations:** 1 Department of Medical Microbiology, University of Groningen, University Medical Center Groningen, Groningen, The Netherlands; 2 Department of Medical Microbiology, University Medical Center Utrecht, Utrecht, The Netherlands

**Keywords:** *Enterococcus faecium*, VRE, Evolution, Diagnostics, Infection control

## Abstract

Early in its evolution, *Enterococcus faecium* acquired traits that allowed it to become a successful nosocomial pathogen. *E. faecium* inherent tenacity to build resistance to antibiotics and environmental stressors that allows the species to thrive in hospital environments. The continual wide use of antibiotics in medicine has been an important driver in the evolution of *E. faecium* becoming a highly proficient hospital pathogen.

For successful prevention and reduction of nosocomial infections with vancomycin resistant *E. faecium* (VREfm), it is essential to focus on reducing VREfm carriage and spread. The aim of this review is to incorporate microbiological insights of *E. faecium* into practical infection control recommendations, to reduce the spread of hospital-acquired VREfm (carriage and infections). The spread of VREfm can be controlled by intensified cleaning procedures, antibiotic stewardship, rapid screening of VREfm carriage focused on high-risk populations, and identification of transmission routes through accurate detection and typing methods in outbreak situations. Further, for successful management of *E. faecium,* continual innovation in the fields of diagnostics, treatment, and eradication is necessary*.*

## Introduction

Enterococci were first discovered in human fecal flora in 1899. However until 1984, they were still considered part of the genus Streptococci [1]. *Streptococcus faecalis* was first described in 1906 when the microorganism was isolated from a patient with endocarditis. *Streptococcus faecium* was first detected in 1919. Later on, streptococci belonging to serogroup D were divided into two groups. This division was made based upon studies demonstrating differences in biochemical and differences from nucleic acid (DNA-rRNA homology studies and 16SrRNA) [2]. *Streptococcus faecalis* and *Streptococcus faecium* were placed in the enterococcus group, which more than 50 species belong [3].

Among the enterococci, *E. faecalis* and *E. faecium* are the main causative agents of infection in humans. In the 1970s, enterococci emerged as a leading cause of hospital-acquired infections [4]. In the past two decades, *E. faecium* has rapidly evolved as a worldwide nosocomial pathogen by successfully adapting to conditions in a nosocomial setting and acquiring resistance against glycopeptides [5, 6]. The resistance genes against glycopeptides are organized in *van* operons located on mobile genetic elements (MGEs). The operons include regulatory genes controlling the expression of ligase genes conferring resistance to glycopeptides, of which the *vanA* and *vanB* genes are the most common [7].

In this review, we will first describe the historical rise of *E. faecium* infections in hospitals worldwide, followed by the subsequent emergence and epidemiological background of vancomycin resistant *E. faecium* (VREfm). Next, we review difficulties in VRE detection and infection control in the modern hospital settings, in which *E. faecium* has emerged as an important pathogen in the past 20 years. Finally, we provide practical recommendations based on these microbiological insights.

### The evolution of *Enterococcus faecium* as a hospital-adapted pathogen

Population genetics and genomics showed that there are two distinct subpopulations of *E. faecium*. The first subpopulation represents commensals of the gastrointestinal (GI) tract and is usually not involved in clinical infection. The second subpopulation represents hospital-associated (HA) *E. faecium* lineages that cause nosocomial outbreaks and opportunistic infections in hospitalized patients. The presence of these distinct subpopulations were recognized two decades ago using amplified fragment length polymorphism (AFLP); a fingerprint-based typing method [8]. Later, sequence-based methods such as multi-locus sequence typing (MLST) and whole genome sequencing (WGS) confirmed and further described these distinct *E. faecium* subpopulations [9–11]. Currently, these two populations are designated as clade A and clade B. The successful HA lineages belong to a subclade of clade A, A1, previously designed as clonal complex 17 (CC-17) [12].

*E. faecium* isolates belonging to the HA subpopulation are characterized by ampicillin resistance and pathogenicity islands; they are also commonly associated with hospital outbreaks [11]. In addition, genome wide studies have shown that these HA isolates acquired a number of traits making them successful in the hospital environment; such as an increase in antibiotic resistance genes and virulence genes enhancing biofilm formation and colonization [13]. These adaptive traits are the result of gene acquisition and gene loss in *E. faecium,* which is facilitated by plasmid transfer, and through homologous recombination, mediated by insertion sequence (IS) elements. IS elements may provide homology at specific sites in the chromosome, allowing for integration of foreign genes by homologous recombination events [10]. The continuous refinement of genomic configuration, characterized by the flux and integration of successful adaptive traits, results in a selective advantage and clonal expansion of HA lineages [14] .

**Fig. 1:**
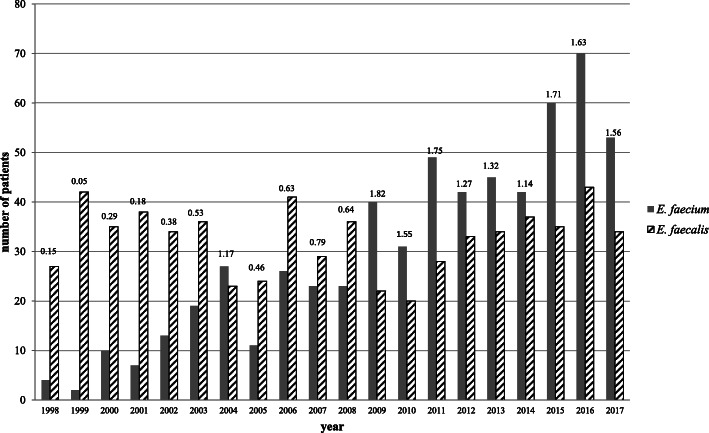
Change in *E. faecium* to *E. faecalis* ratio. Number of patients with blood cultures with *E. faecium* and *E. faecalis* in individual patients and the *E. faecium/E. faecalis* ratio during 1998–2017 in the University Medical Center Groningen. The *E. faecium* to *E. faecalis* ratio changed approximately from 0.1 in 1998 to 1.6 in 2017

In the 2000’s, nosocomial infections with ampicillin resistant *E. faecium* (AREfm) emerged in Europe, replacing *E. faecalis* infections [15]. In fact, European Antimicrobial Resistance Surveillance System (EARSS) data of 2002–2008 showed the largest increase (on average annually 19.3%) in the number of positive *E. faecium* blood cultures compared to the increase of other pathogens as *E. coli, S. aureus, S. pneumoniae* and *E. faecalis* [16]. This emergence of *E. faecium* bloodstream infections (BSI) was also observed in surveillance data of the University Medical Center Groningen (UMCG, The Netherlands). The ratio of positive blood cultures with *E. faecium* and *E. faecalis* in individual patients during 1998–2017 in shown in Fig. 1. While the incidence of *E. faecalis* BSI remained rather constant, the *E. faecium* to *E. faecalis* ratio changed approximately from 0.1 in 1998 to 1.6 in 2017. Hospitals in throughout Europe including Ireland, Spain, Poland, Denmark and Switzerland relate the increase of *E. faecium* bloodstream infections (BSI) to CC-17 clones [17–21]. Furthermore, countries outside Europe also observed increasing infections with *E. faecium*. The United States (US) observed an increase in *E. faecium* BSI from 2002, with a peak in 2010 (prevalence of 5.4%) [22]. An overview of antimicrobial-resistant pathogens causing hospital acquired infections in the US during 2011–2014, showed an overall contribution of *E. faecium* of 3.7% [23], with the highest contribution in catheter-associated urinary tract infections (CAUTI). In 2014, the Australian Enterococcal Sepsis Outcome Program (AESOP) reported that a large proportion (39.9%) of enterococcal bacteremia was caused by *E. faecium* [24].

### Emergence and epidemiology of vancomycin resistant enterococci (VRE)

The acquisition of resistance against glycopeptides is an important landmark in the evolution of enterococci towards a highly resistant microorganism. The first reported (Van-A-type) VRE was in 1988 in France and the United Kingdom [25, 26]. Most VRE outbreaks are due to HA-vancomycin susceptible *E. faecium* (VSEfm) that has acquired the *vanA* or *vanB* gene [27, 28].

**Fig. 2:**
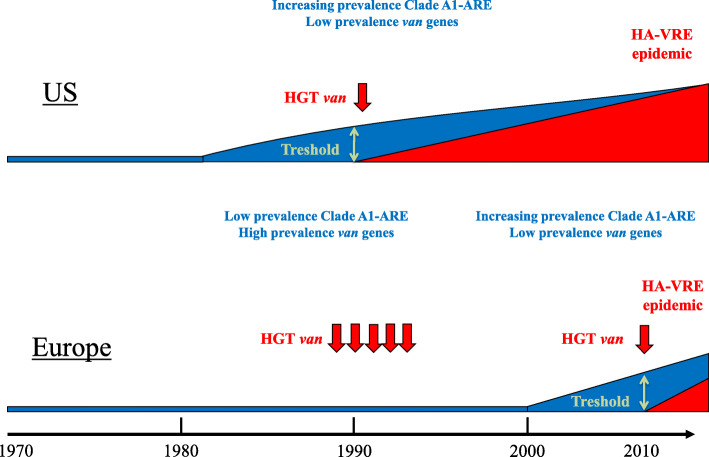
Course of events in the epidemiology of AREfm and VREfm and the differences between the US and Europe from 1970 till 2010. In the United States (US) the increase of AREfm started around 1980 followed by an increase of VRE. In Europe, this event started 20 years later. Note the different situation between the US and Europe; in contrast to the US, Europe did have a large reservoir of VRE in the community in the 1990s, yet without suitable HA AREfm populations in hospitals to take up the *van* genes and become HA VREfm. This reservoir of VRE was linked to the avoparcin use in husbandry. In blue: Hospital Clade A1-VSEfm (AREfm). In red: hospital-Clade A1 VREfm. HGT: horizontal gene transfer (of *van* genes). Threshold: hypothetical critical number of hospital clade A1 AREfm strains needed for the introduction of *van* genes

VanA-type VRE dominated the epidemiology of VRE in the US and Europe [29]. In the US, (*vanA*) VRE had already emerged by the 1990s while remaining rare in hospitals throughout Europe. In both continents, the emergence of AREfm preceded the emergence of VREfm [30]. In Europe, hospital infections with AREfm started to increase from 2000, followed by an increase in VREfm [31], which was comparable to the US 20 years earlier (Fig. 2).

In contrast to the US, Europe had a large reservoir of VRE in the community by the 1990s, yet without suitable HA AREfm populations in hospitals to take up the *van* genes and become HA VREfm. This large reservoir of VRE in the community and farm animals were linked to the avoparcin use in husbandry [28]. Avoparcin, a glycopeptide antibiotic similar to vancomycin, had been in use since 1970 as a growth promotor in the agricultural sector in several European countries. Its use was associated with high numbers of *vanA*-carrying VRE in meat products and samples from livestock [32, 33]. Avoparcin was not used in the US and a community reservoir of VRE was therefore absent [34]. In the US, the rise in VRE was probably due to the extensive use of antibiotics in humans [35] along with failures in infection prevention measures leading to cross transmissions [36]. Because of the potential risk of transmission of VRE or *van* genes from the community into the hospitals, the use of avoparcin was banned in European countries in 1997. As a result, VRE in farm animals declined rapidly. However, persistence of vancomycin resistance in *E. faecium* in poultry farms has been reported in several European countries [37]. It is not known to which extent these mobile genetic elements (MGEs), such as (*vanA*) transposons are still a potential reservoir for HA VREfm [38, 39].

**Fig. 3:**
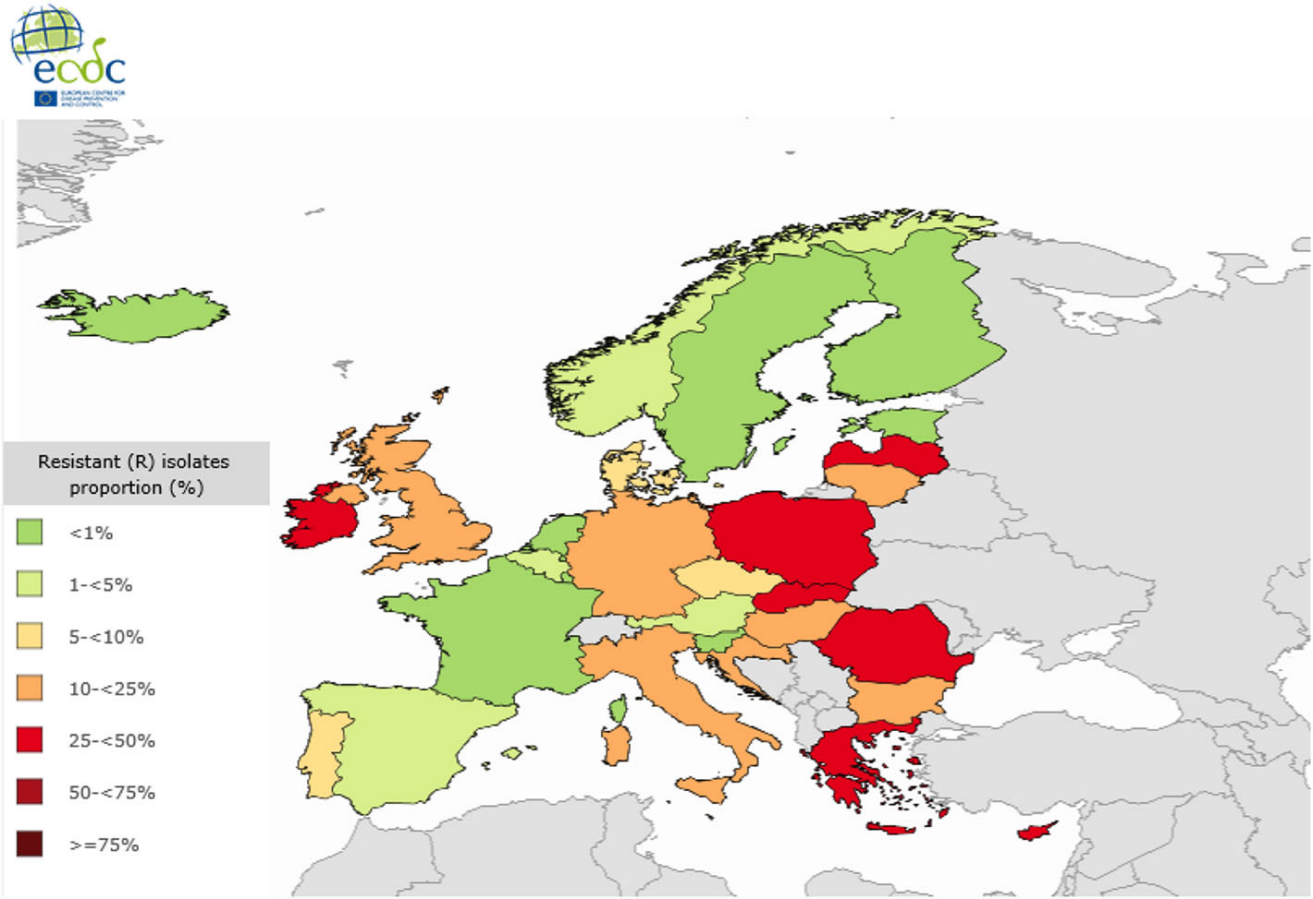
Surveillance data for vancomycin resistant *Enterococcus faecium* in Europe. Data from the ECDC Surveillance Atlas- Antimicrobial resistance. Showing vancomycin resistance proportion rates in *Enterococcus faecium* in Europe for 2016. Dataset provided by ECDC based on data provided by World Health Organization (WHO) and Ministries of Health from the affected countries

**Fig. 4:**
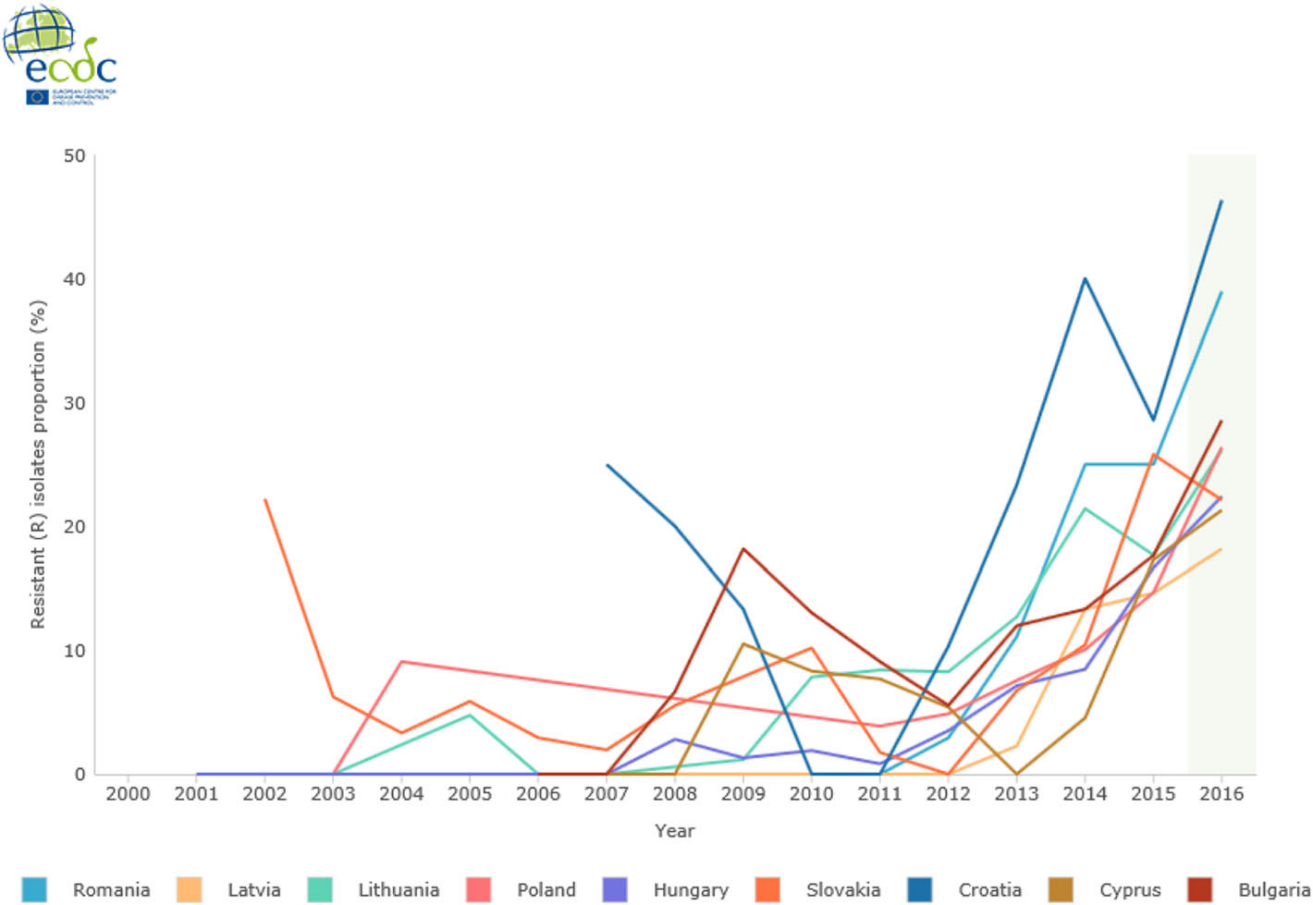
vancomycin resistant *Enterococcus faecium* proportion rates in Eastern European countries from 2002 till 2016. Data from the ECDC Surveillance Atlas- Antimicrobial resistance. Showing the rapid increase in vancomycin resistance proportion rates in *E. faecium* for selected (Eastern) European countries: Romania, Latvia, Lithuania, Poland, Hungary, Slovakia, Croatia, Cyprus and Bulgaria. Dataset provided by ECDC based on data provided by WHO and Ministries of Health from the affected countries

Data collected from 2011 to 2014 by the Centers for Disease Control and Prevention (CDC) about antibiotic resistant hospital acquired infections, showed a high but decreasing prevalence of VREfm in the US, from 80.5% in 2011 to 75.6% in 2014 [40, 41]. Data from the European Center for Diseases and Control (ECDC) for 2016 showed variable surveillance data for VREfm between the European countries [42]. For example, the proportion of VREfm is < 1% in Sweden, Finland, the Netherlands and France; while Cyprus reports the highest proportion of 46.3% (Fig. 3). Notable increases in the proportion of VREfm has occurred in the following Eastern European countries: Romania, Latvia, Lithuania, Poland, Hungary, Slovakia, Croatia, Cyprus and Bulgaria (Fig. 4). The ECDC surveillance Atlas on Antimicrobial resistance reports VREfm proportion rates for these countries in 2016 as follows: Romania 39%, Latvia 28.6%, Lithuania 21.3%, Poland 26.2%, Hungary 22.4%, Slovakia 26.4%, Croatia 22.1%, Cyprus 46.3% and Bulgaria 18.2%. Little is known which lineages and *van*-types are involved in the significant increase of VREfm in these countries.

Importantly, this increase in *vanB* VRE was reported in several European countries around 2005; amongst others in Spain, Greece, Germany and France [43]. A study from Poland investigated the VRE epidemiology from 1999 to 2010 and reported an increasing prevalence of *vanB* VREfm [44–50]. Hospitals in Sweden had a low prevalence of VRE, with *vanB* VRE being detected sporadically. In 2007, outbreaks in three Swedish hospitals occurred and further clonal dissemination from *vanB* VRE were seen [51]. In Germany, the emerge of *vanB* was typically associated with MLST ST192, a lineage within CC-17 [47, 48]. In 2016, the proportion of *vanB* VRE was, for the first time, higher than *vanA* VRE [49]. .Also the Netherlands, reported a the quite significant proportion of *vanB* VRE. Of the 706 VRE strains that were analyzed between May 2012 and March 2016 from 42 Dutch hospitals, 363 carried the *vanA* gene, 340 the *vanB* gene, four carried both the *vanA* and *vanB* gene, and two carried the *vanD* gene [52].

Australia reports an increasing trend in VRE prevalence similar to many countries in Europe. The AESOP and AURA (Antimicrobial Use and Resistance in Australia) reports show a steady increase in VREfm from 36.5% in 2010, to 48.7% in 2015 [24, 53–57]. The latest AURA report of 2017 showed the percentage of VREfm in blood cultures of 2015 ranging from 11.3 to 75% in the different states and territories of Australia [56]. The majority of these isolates were grouped into CC-17. Since 2010 ST203 has had a predominant place across most regions of Australia. Other predominant sequence types are ST17, ST555 and the rapidly increasing ST796, largely replacing ST203 [54]. The emergence of this new clone demonstrates the flexibility of the *E. faecium* genome to continuously respond and adapt to hospital and environmental changes. VanB-type VRE dominated the epidemiology of VRE in Australia, but in recent years VanA-type VRE has emerged. In 2010, *vanA* VREfm was rarely detected compared to 2014, when 18.5% of the VREfm bacteremia isolates harbored the *vanA* gene [58]. The recent emergence of *vanA* VREfm has been associated with several STs and *vanA*-containing plasmids. This suggests multiple introductions of the *vanA* operon into the circulating *E. faecium* clones. This could be due to sources in the community or through introduction by health-care associated travel from overseas [24].

Reports from countries in Asia, South-America, Africa, Russia and the Middle-East [59, 60] about the emergence of VREfm demonstrate the successful spread HA- *E. faecium* lineages worldwide.

In summary, nosocomial VREfm lineages are on the rise in hospitals over all continents. Recent data showed the proportion *E. faecium* clinical isolates that are vancomycin resistant varies between < 1 and 46.3% in Europe, from 75 and 80% in the US, and from 11.3 and 75% in Australia. The incorporation of MGEs such as *vanB*-carrying transposons into successful circulating HA-VSEfm lineages is a significant factor in the emergence of *vanB* VREfm. This occurs via the exchange of large chromosomal fragments, including the transposon Tn*1549* carrying the *vanB* resistance, between *vanB* VREfm and VSEfm [61–66]. Incidentally, de novo acquisition of *Tn1549* from anaerobic gut microbiota to VSEfm may occur [49, 67]. If these events are subsequently followed by clonal expansion, it could lead to an increase in numbers of *vanB* VREfm [68]. However, the success factors for the rapid dissemination of *E. faecium* are probably not limited to the acquisition of antibiotic resistance and virulence genes, but also to specific adaptations to hospital conditions.

### Difficulties to detect and control nosocomial VRE outbreaks

*E. faecium* has to overcome many challenges to remain endemic in hospital environments. The spread of highly resistant microorganisms (HRMOs) in hospitals is generally limited by universal standard precautions and disinfection of patient rooms and medical equipment. In addition, the transmission can be stopped by contact isolation of patients and targeted antibiotic treatment for HRMOs. HRMOs that are undetectable may spread in the hospital and thereby have an advantage over detectable phenotypes. Diagnostic strategies may therefore have a selective role in the emergence of hospital lineages.

In literature, several evasion mechanisms has been reported in VanA-type as well as VanB-type VRE, avoiding detection by the standard recommended methods. The most common standard recommend methods used for detection of glycopeptide resistant enterococci are minimum inhibitory concentration (MIC) determination, disk diffusion, and the breakpoint agar method [68, 69].

Detection of *vanB* VRE can be challenging since routinely measured vancomycin MIC values can range from ≤0.5 mg/L to ≥32 mg/L, as has been shown when using the Vitek2 (bioMérieux) automatic susceptibility testing system [70]. Strains that are *vanB* positive but are determined to be vancomycin susceptible according to the European Committee on Antimicrobial Susceptibility Testing (EUCAST) susceptibility breakpoint of ≤4 mg/L [71, 72], are at risk of spreading without detection. Percentages of these *vanB*-positive low-level vancomycin resistant VRE strains can range from 24.5–55% in hospital outbreak settings [73]. Moreover, the sensitivity of VRE screening declines as the fecal VRE density decreases and if the culture media is assessed at 24 h instead of 48 h [71, 72]. This has led to the suggestion that multiple rectal swabs (up to four or five rectal swabs) are required to detect > 90–95% of the carriers [74]. The direct detection of *vanB* carriage by molecular detection can also be compromised by many false positive results due to *vanB* genes in non-enterococcal anaerobic bacteria present in the gut [75, 76]. An enriched inoculated broth containing metronidazole can be used in a polymerase chain reaction (PCR)-based VRE screening. Additionally, cut-off cycle threshold (Ct)-values can be adjusted to differentiate between detection of *van* genes carried by VRE and interfering signals from the anaerobic flora [77–81].

*VanA* VRE detection can be complicated by variations in the phenotype. *Van* genes are located in an operon that include regulatory genes controlling their expression. The expression of resistance to the glycopeptide teicoplanin can be heterogeneous, corresponding into a VanB-phenotype [82]. The presence of *vanS* (sensor) and *vanR* (regulator) regulatory genes in the *vanA* cassette are essential for the expression of glycopeptide resistance. Some isolates can test vancomycin and teicoplanin susceptible because of major nucleotide deletions or even absence of *vanS* and *vanR* genes in the *vanA* transposon [83] or due to insertion of *IS* elements in the coding regions of the *vanA* transposon [84, 85]. The *vanA*-positive enterococci that are phenotypically susceptible to vancomycin are also termed vancomycin-variable enterococci (VVE) [86]. The VVE are in ‘stealth mode’ and are at risk to spread without detection. In case of major deletions, or complete absence of *vanS/R* genes and thus being non-functional genes, strains will probably not revert under vancomycin therapy. However, in case of small deletions in the *vanR/S* region, or if the region is silenced by *IS* elements, VVE strains can revert into vancomycin resistant strains upon vancomycin therapy [87], which can lead to treatment failure.

VRE may also evade detection by molecular diagnostics because multiple distinct gene clusters may confer resistance to vancomycin. Nine different *van* ligase genes in enterococci have been described (*vanA, B, C, D, E, G, L, M,* and *N*) [86, 88]. Since VRE outbreaks are mainly due to *vanA* and/or *vanB* VREfm [89–92], PCR-based methods most often only target *vanA* and *vanB*, but not the other types of *van* genes. VRE harboring mobile genetic islands with *vanD* are sporadically found in patients, but no dissemination of these islands has yet been detected [28, 93]. However, its prevalence may be underreported since the *vanD* gene is not detected by routine molecular diagnostics.

### Infection control measures

Enterococci are highly tenacious microorganisms by nature. Compared to their ancestors, enterococci acquired traits that have led to an increased tolerance to desiccation and starvation, which make them resistant to environmental stresses similar to modern hospitals [94]. To survive in a hospital environment the adaptive traits of high tenacity and resistance to disinfection procedures are important for the hospital VRE lineages, allowing them to survive for many years in a hospital environment [95]. Enterococci are therefore excellent indicators of environmental contamination [96, 97]. Enterococci are often isolated from high-contact points such as bed rails, over-bed tables, blood-pressure cuffs, alarm buttons, toilet seats and door handles [98]. As a consequence, transmission of enterococci not only occurs directly through contaminated hands of health care workers, patients, or visitors, but also indirectly through contaminated environmental surfaces [99]. Contaminated surfaces represent hidden reservoirs, from which enterococci may re-emerge and colonize patients that are subsequently admitted to the contaminate room [7]. In attempts to eradicate persistent reservoirs with VRE, intensified cleaning measures like targeted cleaning of environmental surfaces using high concentrations of sodium chloride or decontamination with hydrogen peroxide vapor should be used [96, 100].

Enterococci can be tolerant to low concentrations of chemicals such as alcohol and chlorine [101, 102]. After the intensified introduction of alcohol-based hand rubs in Australian hospitals, the use of hand alcohols increased during 2001–2015. When investigating HA *E. faecium* strains isolated from Australian hospitals between 1998 and 2015, there was a significant increase in isopropanolol tolerance over time [103]. Although the alcohol tolerance experiments were established with a concentration of 23%, lower than the 70% which is used in hand alcohols, these tolerant *E. faecium* isolates still survived better than the less tolerant isolates after the 70% isopropanolol surface disinfection. This exemplifies how *E. faecium* can adapt to environmental changes such as an increased use of hand alcohols. Inter-individual differences in hand hygiene compliance between healthcare workers could lead to a variety in VREfm reductions on hands. In case of limited reduction, there might be an unforeseen spread of VREfm.

The characteristic of heat-resistance is an important adaptive trait of enterococci*.* In early studies, the exceptionality of heat-resistance in enterococci had been reported in investigating pasteurization of dairy products [104]. A study comparing heat resistance of VSE versus VRE showed that some vancomycin-resistant isolates even survived exposure to 80 degrees Celsius for several minutes [105]. This is of particular relevance for infection control practices, since disinfection procedures of bedpans regularly include heating at 80 degrees for 1 min.

Several infection prevention strategies have been advised by the CDC Hospital Infection Control Practices Advisory Committee (HICPAC) for controlling VRE such as; prudent use of vancomycin, education programs for hospital staff, early detection and reporting of VRE by clinical microbiology laboratories, and isolation precautions and implementation of infection-control measures to prevent transmission of VRE including contact isolation for VRE-positive patients [103]. It is difficult to state which infection prevention measure by itself has the highest impact. The implementation of hand hygiene and decreasing environmental contamination by cleaning measures have a significant impact on reducing the spread of VRE [106]. However, single infection prevention measures often fail to have a real effect on reducing VRE rates. A multifaceted program implementing several guidelines, such as advised by the HICPAC, are therefore often needed to observe a clear reduction in VRE rates [107, 108].

Antibiotic use, especially metronidazole, vancomycin and cephalosporins are risk factors for VRE acquisition [109, 110]. Treatment with antibiotics that have activity against anaerobic bacteria can lead to a profound proliferation of VRE in the GI tract and subsequent BSI [111–114]. Ceftriaxone usage has also been associated with VRE BSIs [111, 115]. This demonstrates that the stringent use of antibiotics to reduce the selective pressure is important and has successfully been applied in controlling ongoing VRE outbreaks [116].

Since a patient with an infection caused by VRE could be the tip of an iceberg [117, 118] active surveillance is needed to detect VRE-carriage in patients in high-risk units [119]. Screening patients transferred from foreign countries with high VRE prevalence is also another important infection prevention measure.

### Molecular typing of VRE

In VRE outbreak investigations, rapid and accurate typing is required to investigate the genetic relatedness between patients’ isolates. This information is essential to demonstrate nosocomial transmission and whether it is needed to enhance infection prevention measures. Rapid typing followed by infection prevention measures can lead to rapid control of nosocomial spread [75]. In Table 1, we summarized common used VRE typing methods and accompanying characteristics; reproducibility, ease of performance, data interpretation, ease of data exchange and costs.

**Table 1:** Vancomycin resistant enterococci typing methods and accompanying characteristics

Method	MLVA	MLST	PFGE	cgMLST	WGS	Transposon analysis
Principle	Fragment length of variable tandem repeat loci	Sequences of multiple house keeping genes	DNA based macro restriction analysis	Genome-wide gene-by-gene approach of 1423 genes on allelic level	Whole genome analysis	Sequences of transposon content and integration
Reproducibility	High	High	Medium	Excellent	Excellent	Excellent
Ease of performance	Very easy	Easy	Laborious	Easy	Easy	Easy
Data interpretation	Easy-moderate	Easy	Difficult	Easy	Various	Moderate
Ease of data exchange	Easy	Easy	Difficult	Easy	Possible	Possible
Costs	Low	Medium	Medium	High, extracted from WGS	High	High, extracted from WGS
Discriminatory power	Low	Medium	High	Excellent	Excellent	Additional

*MLVA* Multiple Locus Variable Number of Tandem Repeat Analysis, *MLST* Multi-locus Sequence Typing, *PFGE* Pulsed-field gel electrophoresis, *cgMLST* core-genome MLST, *WGS* whole-genome sequencing

WGS is increasingly used in (VRE) outbreak analysis [120] and provides the highest discriminatory power. In addition, WGS offers the possibilities to perform pan-genome analysis to even enhance the assessment of genetic relatedness [48, 121, 122]. Additionally, a wide range of information can be extracted from WGS data such as MLST, core-genome (cg) MLST, whole-genome (wg) MLST data, virulence factors, resistance genes, plasmids and other genetic markers. However, there are some challenges to overcome to make it more accessible in daily routine clinical microbiology and outbreak analysis. Most important are the standardization and validation of procedures [123] and the interpretation of data [124]. The ease of data interpretation depends on the type of analysis to perform and which tools are available [125]. For example, cgMLST data can easily be extracted from WGS data by several in-house and commercially software packages. Compared to MLST, cgMLST has a higher discriminatory power in distinguishing genetically related and unrelated *E. faecium* isolates [126–128]. The advantage of cgMLST over single-nucleotide polymorphism (SNP)-based methods is that the data can easily be compared, stored and shared in web-based databases that can be interrogated (http://www.cgmlst.org/ncs/schema/991893/). Importantly, if VRE outbreaks are caused by horizontal transfer of MGEs encoding vancomycin-resistance, studying the molecular epidemiology of these MGEs by specifically analyzing the various transposons encoding *vanA* or *vanB* gene clusters is essential. The use of WGS facilitates detailed analysis of variation in these transposons. These transposon analyses will enhance the resolution of used typing methods and provide better insight in VRE outbreaks [129].

## Conclusion and future perspectives

In the future, it will be a challenge to withstand the spread of VREfm. A rapid and ongoing emergence of VREfm has been observed in countries in Central and Eastern Europe. Variances within same countries along with large regional differences have been observed in this rise of VREfm infections. This is underlined by the regional differences in VREfm proportions in German and Dutch regions. In 2016, the lowest proportion in Germany was reported in the region of North-West Germany (5.9%), which is in contrast with the proportion in the North-East (9.5%), South-East (16.2%), and South-West (17.6%) [68, 69]. The proportion of VRE in the Dutch Northern-East region bordering with North-West Germany remained very low between 2013 and 2016. Among these two regions, collaborative cross-border INTERREG-projects focusing on prevention of the spread of highly-resistant microorganisms are ongoing. Although there is no conclusive explanation for the variations in the German regions, surveillance and outbreak management strategies, antibiotic stewardship policies [130], and differences in patient traffic from high prevalence countries may be important factors. In some countries, VRE infection control policies focuses only on patients with infections, while other countries patients belonging to high-risk populations are also screened for VRE-carriage as recommended by HICPAC [131]. VRE infections are commonly preceded by VRE-carriage, as described in our review. Early detection of carriage may prevent the spread and reduce the number infections. In the Netherlands there have been many outbreaks with patients carrying VRE. These outbreaks were controlled in an early phase, and thereby the proportion of infections with VRE is still low in the Netherlands.

Thus, if the goal of a hospital is to prevent VREfm infections, special attention is required to reduce the VREfm spread by screening for VREfm-carriage. Other important factors are the role of hospital environment contamination by VREfm and the challenges in detection and typing of VREfm. We summarize recommendations described in literature and/or by guidelines in Table 2. Many of the recommendations follow directly from the microbiological traits of *E. faecium* as we reviewed. So far, these recommendations have shown to be successful in the control of VREfm in the Netherlands. However, these measures are very expensive and require a lot of effort from medical (molecular) microbiologists and infection control specialists [106]. Adequate VREfm diagnostics and typing can be difficult, as described in this review. Innovations in the detection and typing of VREfm are required to overcome these difficulties. Development of better selective media, PCRs with higher specificity, or rapid point of care tests are needed to detect VREfm more efficiently. A promising development is the use of clone-specific PCRs, which might be helpful to detect and control VREfm outbreaks caused by specific clones [118]. This method combines typing and detection in a rapid and cost-effective manner [138].

**Table 2:** Recommendations for infection control and detection methods of VRE

Traits of *Enterococcus faecium*	Implications for infection control	Recommendations
High tenacity and intrinsic resistance to environmental stress	- Prolonged survival in hospital environment. - High survival to desiccation and starvation. - Resistance to heat and disinfection procedures.	- Intensified cleaning procedures, including intensified cleaning procedures and prolonged disinfection procedures [132]. - Implementation of infection-control measures to prevent transmission of VRE, including isolation precautions for VRE-positive patients [97, 101, 103]. - Education programs for hospital staff, including hand hygiene to prevent further transmission [106]. - Environmental cultures in (uncontrolled) VRE outbreaks and surveillance cultures after disinfections.
Intrinsic resistance antibiotics	- Outgrowth under antibiotic pressure. - Prone to become pan-resistant.	- Antibiotic stewardship, especially prudent use of vancomycin (reduce emergence of VRE) [106] and metronidazole (reduce outgrowth of VRE) [106]. - Surveillance and controlling of VRE-carriage in hospitals [133, 134].
Genome plasticity	- Continuously adaptation and refinement in response to environmental changes. - Development of resistance to newer antibiotics and disinfectants in the future.	- Continuous awareness and surveillance to detect resistance to newer antibiotics and disinfectants. - Further research and development of antimicrobial targets for the treatment of MDR *E. faecium* [106].
Diagnostic evasion	- Phenotypes of evolutionary successful HA VRE lineages that evade detection by standard recommended methods for detection of glycopeptide resistance in *E. faecium* - Difficulties in detecting VRE-carriage due to low fecal densities	- Active surveillance cultures to detect VRE-carriage in patients at high-risk units and patients transferred from foreign countries with high VRE prevalence [135]. - Multiple rectal samples (four to five), are needed to detect the majority of carriers (> 90–95%) [106]. - Get knowledge of the local epidemiology of VRE and vancomycin MICs in own hospital. - Early and accurate detection and reporting of VRE by clinical microbiology laboratories [75, 76]. - For rapid screening of VRE carriage, a combination of selective enrichment broths and molecular detection increases the sensitivity [106]. - Use of selective (chromogenic) agar in the laboratory detection of VRE [82]. - Vancomycin disk diffusion according to EUCAST in the detection of vancomycin-resistance in VRE [136]. - Genotypic testing of invasive vancomycin-susceptible enterococci by PCR [137].
Common origin of hospital lineages in early twentieth century (CC-17)	- Typing difficulties during VRE outbreaks.	- Rapid and accurate typing is needed to take adequate infection prevention measures in VRE outbreaks. - Preferably a highly discriminatory typing method like cgMLST or WGS, ideally combined with transposon analysis should be used in VRE outbreak analysis.

It is a point of debate whether these efforts are worthwhile to control the spread of VREfm. The attributable mortality of the currently successful VREfm lineages is mainly due to inappropriate (empirical) antibiotics rather than additional virulence of vancomycin resistance [139]. However, treatment options are limited in VREfm, since *E. faecium* is intrinsically resistant to many antibiotic classes. Resistance to several last-line enterococcal drugs like linezolid, daptomycin, tigecycline, and quinopristin-dalfopristin have already emerged [140–142]. Further research and development of antimicrobial targets is needed for the treatment of multidrug resistant (MDR) *E. faecium* [143–146]. Development of new antibiotics is expensive, requires time, and has a risk of rapid development of resistance to these new drugs. Therefore, it is important to use the current available antibiotics prudently and optimize adherence to hygiene precautions to prevent the patient-to-patient spread of VREfm resistant to these last-line antibiotics. It may be wise to reduce the spread of VREfm by surveillance in high risk populations. However, in many hospitals this might be difficult to realize. Capacity building programs and structural financial support for hospitals would be needed to implement efficient nosocomial screening for VREfm-carriage and subsequent infection control measures. Cross-border collaborations may prove useful in the implementation of such programs and have previously shown to be successful in decreasing the methicillin resistant *Staphylococcus aureus* (MRSA) prevalence in the Dutch-German Euregion [135].

## Data Availability

All data generated or analysed during this study are included in this published article.

## Abbreviations

AESOP: Australian Enterococcal Sepsis Outcome Program
AREfm: Ampicillin resistant *E. faecium*
AURA: Antimicrobial Use and Resistance in Australia
BSI: Bloodstream infections
CC-17: Clonal complex 17
CDC: Centers for Disease Control and Prevention
Ct: Cycle threshold
EARSS: European Antimicrobial Resistance Surveillance System
EUCAST: European Committee on Antimicrobial Susceptibility Testing
GI: Gastrointestinal
HA: Hospital adapted
HICPAC: Hospital Infection Control Practices Advisory Committee
HRMO: Highly resistant microorganism
IS: Insertion sequence
MDR: Multidrug resistant
MIC: Minimum inhibitory concentration
MGE: Mobile genetic element
MLST: Multi-locus sequence typing
cgMLST: core-genome MLST
wgMLST: whole-genome MLST
MLVA: Multiple Locus Variable Number of Tandem Repeat Analysis
MRSA: Methicillin resistant *Staphylococcus aureus*
PFGE: Pulsed-field gel electrophoresis
PCR: Polymerase chain reaction
SNP: Single-nucleotide polymorphism
UMCG: University Medical Center Groningen
US: United States
VRE: Vancomycin resistant enterococci
VREfm: Vancomycin resistant *E. faecium*
VSEfm: Vancomycin susceptible *E. faecium*
VVE: Vancomycin-variable enterococci
WGS: Whole genome sequencing
WHO: World Health Organization
